# Accuracy of Robotic Surgical Assistant (ROSA®) Surgical Plan in Total Knee Arthroplasty

**DOI:** 10.7759/cureus.97289

**Published:** 2025-11-19

**Authors:** Vera T.J. Schriebl, Henriëtte M. Eijking, Isobel M. Dorling, Emil H. Van Haaren, Roel Hendrickx, Martijn G.M. Schotanus, Jasper Most, Bert Boonen

**Affiliations:** 1 Department of Orthopedic Surgery, Zuyderland Medical Center, Sittard-Geleen, NLD; 2 Department of Orthopedic Surgery, Institute of Nutrition and Translational Research in Metabolism (NUTRIM), Maastricht University, Maastricht, NLD; 3 Department of Clinical Engineering, Faculty of Science and Engineering (FSE), Maastricht University, Maastricht, NLD; 4 Department of Orthopedic Surgery, Care and Public Health Research Institute (CAPHRI), Maastricht University, Maastricht, NLD; 5 Department of Epidemiology and Public Health, Care and Public Health Research Institute (CAPHRI), Maastricht University, Maastricht, NLD

**Keywords:** accuracy, duration of surgery, functional alignment, implant size, robotic-assisted surgery, robotic surgical assistant, total knee arthroplasty (tka)

## Abstract

Introduction: Robotic assistance in total knee arthroplasty (TKA) promises better outcomes through data-supported, personalized alignment. The primary goal of this study was to assess the accuracy of surgical plans delivered by the Robotic Surgical Assistant (ROSA®) Knee System. The secondary goal was to evaluate the variability in knee balancing and implant alignment and to assess the effect of ROSA® on surgery duration and complication rates as compared to the conventional TKA cohort.

Materials: Inclusion criteria were primary TKA indicated by osteoarthritis (OA), age of 40-90 years, body mass index (BMI) of 18.5-50 kg/m^2^, and American Society of Anesthesiologists (ASA) I-III. Surgery was performed by surgeons with at least 10 ROSA® surgeries during the study duration. The primary study parameters were accuracy in femoral and tibial resection levels, assessed by Bland-Altman analysis, implant sizes, and final knee alignment.

Results: The accuracy of medial and lateral distal femoral resection levels was high (mean differences between the plan and validation, -0.50 ± 0.91 mm and -0.59 ± 0.96 mm, respectively). Accuracy of the medial and lateral tibia was less with greater variability (-0.44 ± 2.28 mm and 2.50 ± 3.13 mm, respectively). The femoral component size was accurately predicted in 75% of cases, with overestimation occurring in 20% of cases. Surgical steps were repeated in 59% of femoral cuts and 61% of tibial cuts. Surgical duration was significantly different between groups. ROSA TKA surgery was 18 minutes longer and was prolonged by five minutes per repeated surgical step. The hip-knee-ankle (HKA) axis was 1.6°±1.9° varus post-operatively. Complication rates were not significantly different from ROSA®-assisted TKA as compared to conventional TKA.

Discussion: ROSA®-assisted TKA planning demonstrated a high degree of accuracy for the femur, but a lesser degree for the tibia. Predicting implant sizes was highly accurate. With ROSA®-assistance, alignment differed from mechanical alignment. Future studies should provide a higher level of evidence and investigate the relation between anatomical outcomes and clinical outcomes.

## Introduction

Approximately 20% of patients are not satisfied with the outcomes of primary total knee arthroplasty (TKA) and experience pain, instability, or limited range of motion [1]. Part of this rate is believed to result from inappropriate alignment and imbalance of the TKA [2,3]. Modern technology applied during surgeries is designed to improve implant positioning and alignment by providing data-supported surgery plans and by assisting cuts through jigs and optometric monitoring.

One of the latest advancements in robotic assistance is the Robotic Surgical Assistant (ROSA®) Knee System, supporting alternative alignment strategies to minimize releases of the soft tissue envelope of the knee. The first studies of the ROSA® Knee System show high accuracy of resection (<1.0 mm) from preliminary or cadaveric studies, and confirm deviations from mechanical alignment of >1°, illustrating this shift towards more personalized alignment strategies [4-7]. Clinical data suggests a reduction in outliers, reduced short- and long-term complications, as well as improved patient-reported outcome [8-10]. Data on the necessity for recuts, its effects on surgery duration, and the validity of the validation steps of the robot have not been published.

Therefore, this study validated the accuracy of the ROSA® Knee System surgical plans as compared to their final ROSA®-validated execution. It was hypothesized that ROSA® Knee System surgical plans are highly accurate as compared to final levels of resection and implant sizes. The second aim of this study was to assess variability in knee balancing and alignment. Lastly, we assessed the effects of ROSA® on surgery duration and complication rates in a cohort of conventional total knees operated on during the same period and by the same surgeons.

## Materials and methods

Study design

The first part of the study was a retrospective observational study on the validation of the accuracy of the ROSA® Knee System surgical plans as compared to their final ROSA®-validated execution. The second part of this study was to assess variability in knee balancing and alignment. Lastly, we assessed the effects of ROSA® on surgery duration and complication rates in a cohort of conventional total knees operated on during the same period and by the same surgeons. This study was approved by the local medical ethical committee (Z2022186).

Study participants

Patients were selected from the Excellence Center for Hip- and Knee Arthroplasty at Zuyderland Medical Center in the Netherlands from May 2021 until September 2022. All surgeries were performed by three high-volume orthopedic surgeons (>50 TKAs/year) who performed at least 10 ROSA®-assisted surgeries. The inclusion criteria for patients in this study were defined as follows: scheduled for primary total knee arthroplasty due to end-stage knee osteoarthritis (OA) as defined by Kellgren-Lawrence stages, aged between 40 and 90 years, body mass index (BMI) ranging from 18.5 to 50 kg/m2, and classified as American Society of Anesthesiologists (ASA) Class I-III. No restrictions were taken on the type of deformity (varus or valgus) to ensure a representative AO cohort in our practice. There were no criteria to decide on robotic-assisted TKA or conventional TKA. The decision was up to the surgeons' and patients' preference. No exclusion criteria (e.g., BMI, deformity, age, etc.) were used in any of the two groups to prevent introducing bias. Patients with indications other than end-stage knee OA for TKA were excluded from study participation.

Study parameters

Data were retrospectively collected from electronic patient records and from the ROSA® Knee System. The accuracy of surgical planning was defined by differences in resections between planned and final validation. Validation was measured post-surgically by the ROSA® Knee System. Resections were measured at the lateral and medial distal femur and the lateral and medial proximal tibia in millimeters. Accuracy of resections was defined as a mean difference of 1.0 mm or less and a standard deviation (SD) of 2.0 or less [11]. Posterior femoral condyle resections were not available in the surgery reports and were therefore not assessed. 

Secondary parameters included number of repetitions of surgical step to achieve valid resection for final implantation, number of over- and underestimation of predicted implant size as measured by comparing the planned femoral component sizes to the validated femoral component sizes, final knee alignment as measured by the ROSA® Knee System (hip-knee-ankle (HKA) axis), degrees varus (-) or valgus (+) of individual components, balancing of the TKA and laxity assessment (laxity of the medial collateral ligament (MCL) and lateral collateral ligament (LCL) in extension and 90 degrees flexion after implantation of the definitive implant and before soft tissue closure (targeted laxity was defined as <2 mm and <5 mm and assessed by ROSA® Knee system), duration of surgery (incision to end of surgery as defined after wound closure), blood loss, and complications [12].

Study interventions

The ROSA® Knee System was used in all ROSA®-assisted TKA cases. Two types of implants were used: the Persona (n = 30) and Vanguard (n = 41). Two types of femoral components were used: cruciate retaining (CR) or posterior stabilized (PS). During the study, daily practice for TKA changed from primary implant Vanguard to Persona. There were no criteria used for the type of implant. All ROSA®-assisted TKAs were supported by an engineering consultant from Zimmer Biomet. Primarily, imageless planning for the ROSA® Knee system was used 94% (67/71). There were no criteria set for image-based or imageless cases. The image-based cases had standardized pre-operative radiographs with which a pre-operative plan was created. This plan could be adjusted during surgery. For the imageless cases, an operative plan was made intraoperatively. In both imageless and image-based cases, restricted inverse kinematic alignment was aimed for. Manual stability testing to determine laxity was performed by the surgeon during surgery. Re-resections were performed if laxity was too tight and were done before the final laxity evaluation. Conventional TKA was performed using conventional alignment jigs with the purpose of obtaining a neutral mechanical alignment. There were no criteria to decide on ROSA®-assisted TKA or conventional TKA. The decision was up to the surgeons' and patients' preference. No exclusion criteria were used in any of the two groups to prevent introducing bias. All surgeries were performed using a medial parapatellar approach.

ROSA®-assisted procedure

The ROSA®-assisted TKA procedures are performed as described by the eight-step technique by Eijking et al. [13]. Standard tibial and femoral landmarks were taken, adhering to the protocol. Target alignment is based on 1-2 mm laxity medially in extension and flexion, and laterally 1-3 mm in extension and 2-5 mm in flexion. The laxity of the medial collateral ligament and lateral collateral ligament was measured in extension and at 90 degrees of flexion after final implant insertion prior to soft tissue closure. The robotic arm positioned the cutting jig according to the alignment in the surgical plan. Thereafter, the resections were performed with the assistance of the robot, and finally, validation measurements of resections were performed by the ROSA® Knee system. Soft tissue release was avoided, if possible. In severe, contract cases, necessary releases were documented. All ROSA®-assisted procedures aimed to achieve restricted inverse kinematic alignment: the following alignment boundaries were applied: tibial varus ≤3 °, tibial valgus <0 °, femur varus ≤3 °, femur valgus ≤2 °, and no internal femoral rotation [13].

Statistical analysis

All statistical analyses were performed using IBM SPSS Statistics for Windows, Version 29 (Released 2022; IBM Corp., Armonk, New York, United States). Patient characteristic data were described as mean with standard deviation (normally distributed) or median and interquartile range (not normally distributed) for continuous outcomes and as frequency (in %) for categorical outcomes. For accuracy comparison, Bland-Altman plots were created, and bias and proportional bias by linear regression were assessed with 95% confidence intervals to determine the significance of a factor. A Student’s T-test or Mann-Whitney U-test for normally or non-normally distributed data, respectively, was performed for comparing ROSA® to conventional TKAs. Chi-square tests were used to compare categorical outcomes.

## Results

Patient characteristics

A total of 84 patients were included in the ROSA®-assisted TKA group and 134 patients were included in the conventional group (Table 1). Between the ROSA®-assisted and conventional groups, no differences were observed for age, sex, BMI, and blood loss. Compared to the conventional group, the surgery time of the ROSA®-assisted group was significantly longer (1:24 ± 0:16 hours versus 1:06 ± 0:15 hours; p < 0.001), with no meaningful difference (<5 minutes) between the learning phase and the proficiency phase.

**Table 1:** Demographic and surgical characteristics for the ROSA® and conventional group ROSA: Robotic Surgical Assistant; TKA: total knee arthroplasty The data were represented as mean ± standard deviation. Student’s T-test was performed for comparing groups for continuous outcomes, and the Chi-square test was used for comparing groups for categorical outcomes. p-value was considered significant if p < 0.05

	ROSA® TKA				Conventional TKA				
	N	Mean	SD		N	Mean	SD	P-value	T/Chi^2^
Age (year)	84	73	±9		134	71	±8	0.08	-1.8
Sex (% female)	55	65%			87	65%		0.9	0.01
Length (cm)	83	168.1	±8.1		133	170.0	±9.4	0.14	-1
Weight (kg)	83	83.3	±15.2		133	86.5	±19.2	0.2	-1.3
Body mass index (kg/m^2^)	83	29.4	±4.6		133	29.8	±5.6	0.6	-0.6
Blood loss (mL)	84	283	±126		134	281	±142	0.9	0.1
Surgery duration (h:min)	84	1:24	±0:16		133	1:06	±0:15	<0.001	8.1
Surgeon A	40	1:16	±0:15		44	1:04	±0:18	0.002	3.2
Surgeon B	16	1:27	±0:14		39	1:03	±0:12	<0.001	5.7
Surgeon C	28	1:34	±0:13		50	1:10	±0:13	<0.001	7.6
Kellgren-Lawrence stages								0.58	1.1
Kellgren-Lawrence 2 (%)	1	1%			1	1%			
Kellgren-Lawrence 3 (%)	39	46%			53	40%			
Kellgren-Lawrence 4 (%)	44	52%			79	59%			

Accuracy resections

Due to errors in saving surgical reports, 13 out of 84 (15%) ROSA®-reports were unavailable. Planned and post-operatively measured validation of resection levels for 71 cases is provided in Table 2. On average, all components’ post-operatively measured validations of resections were smaller than planned. All resections were accurate on average, as defined by a deviation between validated and planned resection of <1.0 mm, except for lateral tibial resection. The medial and lateral distal femur resections were precise, as defined by a standard deviation of the difference between planned and validated resections of <1.0 mm. Tibia resection was less precise (SD: >1.0 mm; medial, 2.28 mm; and lateral, 3.13 mm).

**Table 2:** Mean values of the planned, validated, and differences between planned and validated resections Resection data are represented as mean ± standard deviation or confidence interval (for difference). Resections were considered accurate as mean difference of ≤1.0 mm and standard deviation of ≤2.0

	Planned	Validated	Difference
Distal femur			
Medial, mm	8.71 ± 1.44	8.20 ± 1.28	-0.50 ± 0.91 (-2.30-1.29)
Lateral, mm	7.37 ± 186	6.78 ± 1.83	-0.59 ± 0.96 (-2.47-1.30)
Proximal tibia			
Medial, mm	8.15 ± 1.34	7.71 ± 2.02	-0.44 ± 2.28 (-4.91-4.02)
Lateral, mm	6.77 ± 1.82	9.28 ± 2.61	2.50 ± 3.13 (-3.62-8.63)

**Figure 1:**
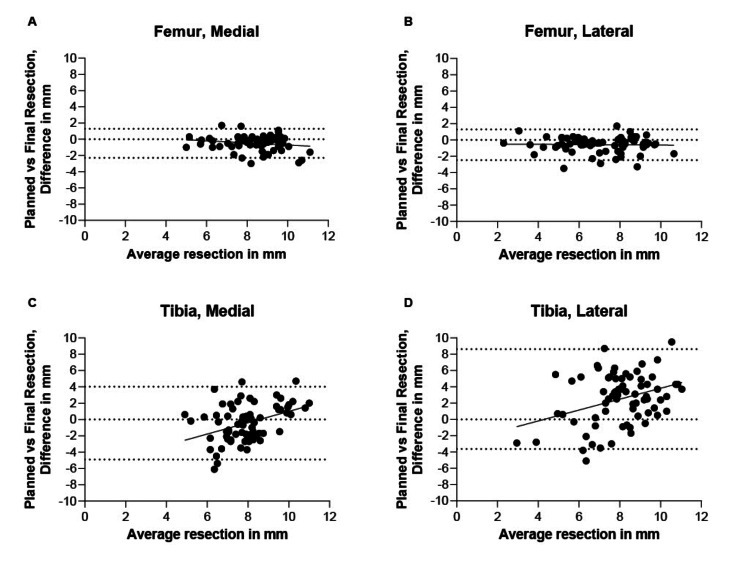
Linear regression of Bland-Altman for the distal femur medial and lateral resection and the proximal tibia medial and lateral resection A: distal femur medial resection; B: distal femur lateral resection; C: proximal tibia medial resection; D: proximal tibia lateral resection Bias (mean difference) and proportional bias (slope average versus difference) were assessed by linear regression of Bland-Altman plots, a 95% confidence intervals were used. p-value <0.05 was considered statistically significant

In the femoral components, no proportional bias was observed (p = 0.14 and p = 0.82, Figure 1). For the tibial resections, planning was less accurate for small and large resections as compared to average resections (for both, proportional bias of 0.7 ± 0.2 per mm resection, p < 0.005, Figure 1).

Implant prediction

The femoral implant component type (CR versus PS) was accurately planned in 71% (n = 50/70) of cases, and size in 75% (n = 50/67) of cases. In 29% (n = 20/70) of cases, a different implant type was used (CR versus PS); in 20% (n = 14/67), the size was overestimated, and in 4% (n = 3/67), underestimated. In total, 58% (n = 39/67) of cases were accurately predicted in type and size.

Surgical steps

**Figure 2:**
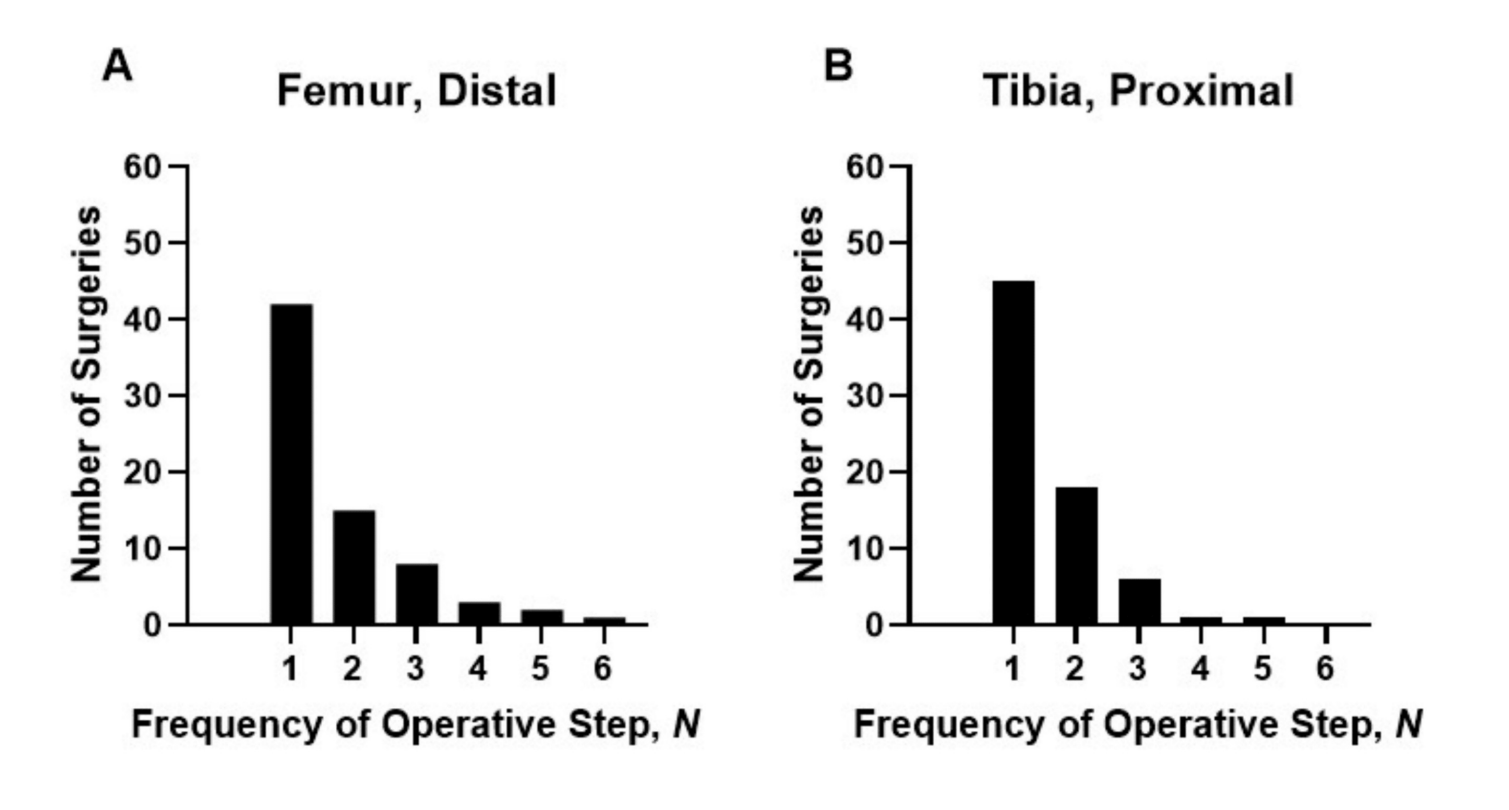
Number of frequencies of operative steps per surgical step A: number of frequencies of operative steps per surgical step for the distal femur; B: number of frequencies of operative steps per surgical step for the proximal tibia

In 59% (n = 43/71) of surgeries, the initial planning and execution of the femoral distal resection did not yield a satisfactory result; hence, resections required at least one repeat in the surgical step necessary (Figure 2). Comparably, in 61% (n = 45/71) of surgeries, proximal tibial resection required at least one repetition.

The number of steps necessary for accurate placement significantly affected surgery duration. For every required repetition in the distal femoral planning, surgery lasted 5.4 ± 2.3 minutes longer (p = 0.02). For the proximal tibia, repetitions were not associated with a significant extension of surgery (+1.8 ± 2.6 minutes; p = 0.5).

Alignment

**Figure 3:**
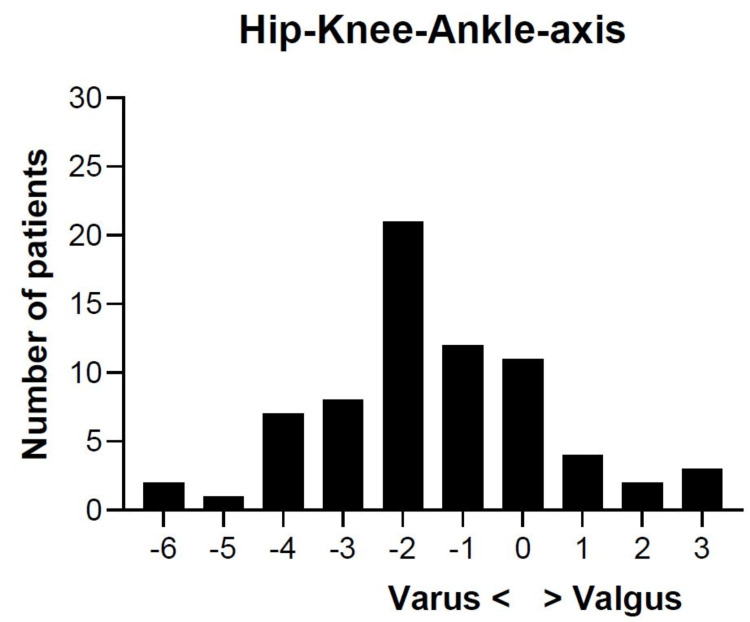
Hip-knee-ankle axis 0 degrees: mechanical alignment; negative value: varus alignment; positive value: valgus alignment Mean and standard deviation of the mechanical, varus, and valgus hip-knee-ankle axis were measured for each patient and categorized for each value

ROSA®-assisted surgeries produced an average deviation from mechanical alignment of the HKA axis (0°) of 1.6° ± 1.9° varus (Figure 3).

Balancing

**Figure 4:**
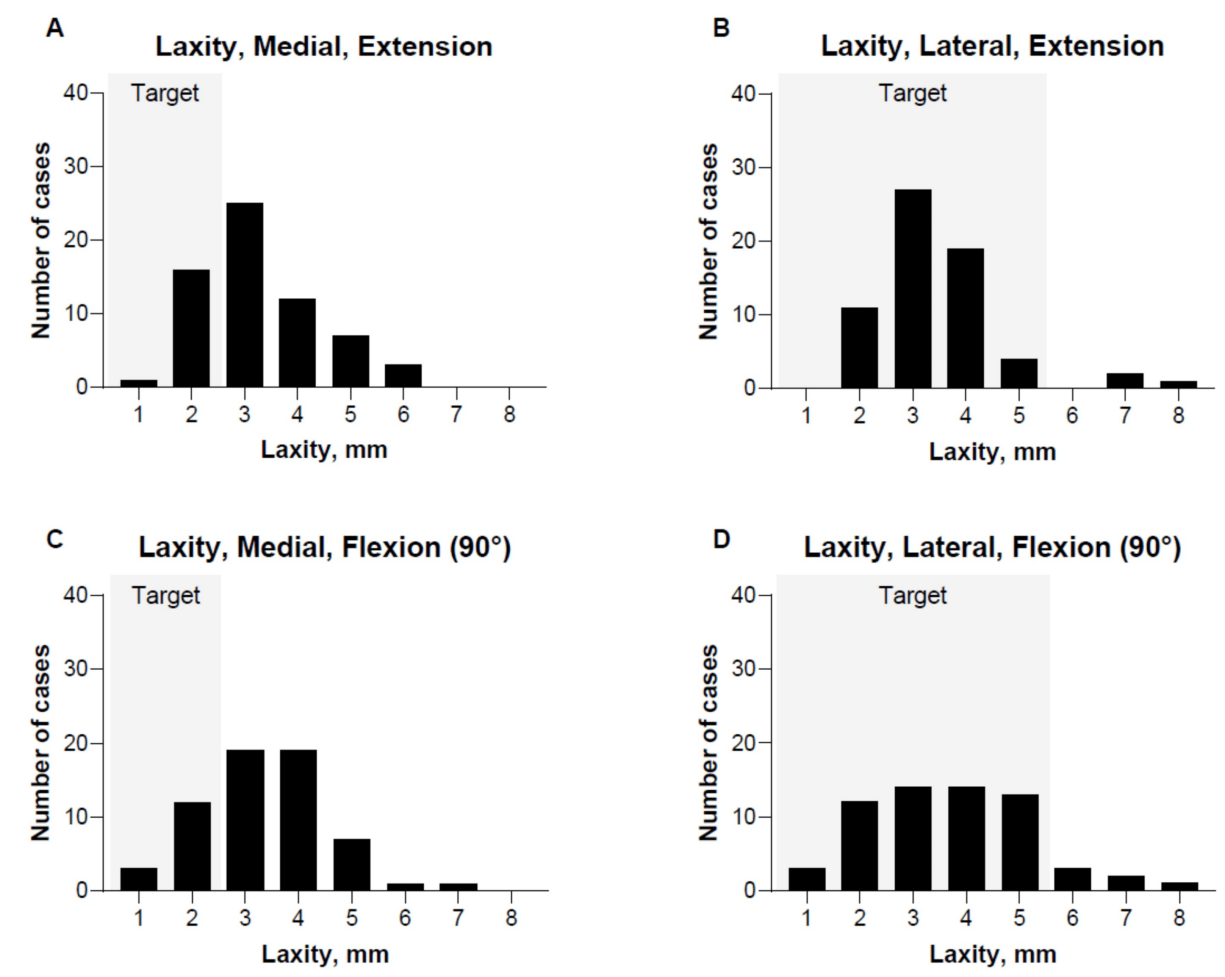
Final medial and lateral collateral ligament laxity A: medial laxity in extension; B: lateral laxity in extension; C: medial laxity in 90 degrees flexion; D: lateral laxity in 90 degrees flexion Laxity after final implant insertion prior to soft tissue closure. Measured in both extension and 90 degrees flexion. The mean and standard deviation were measured for medial laxity in extension and 90 degrees flexion and for lateral laxity in extension and 90 degrees flexion

Laxity assessment of the MCL and LCL was available for 64 cases. The laxity of the medial collateral ligament and lateral collateral ligament is shown in Figure 4. Mean medial laxity of the MCL in extension was 2.81 ± 1.09 mm, and in 90 degrees flexion was 3.12 ± 2.35 mm. For the LCL, the mean lateral laxity in extension was 3.03 ± 1.16 mm, and in 90 degrees, flexion was 3.42 ± 2.12 mm (Figure 4). For medial assessments, 27% (n = 17/64) and 25% (n = 16/64) of cases were measured as ≤2 mm, as targeted, while for lateral assessments, 95% (n = 61/64) and 89% (n = 57/64) of measurements were within target (≤5 mm).

Complications

We observed no difference in the complication rates of ROSA®-assisted versus conventional surgeries (the most frequent complication was wound leakage; 5% (n = 17/64) in ROSA® versus 9% (n = 12/134) in conventional). One ROSA®-assisted TKA patient developed a deep wound infection, whereby reoperation was necessary. No other revision surgery was necessary, and no deaths were reported within the observation period of three months after surgery.

## Discussion

The aim of this study was to validate the accuracy of the ROSA® Knee System for the first time in vivo. We established that levels of resection are accurate in the femur, but less so in the tibia. Secondly, the ROSA® Knee System accurately predicted femoral implant size in three-quarters of the cases. Repetitions of surgical steps due to insufficient resections (up to five repetitions) were required in more than half of the cases per component and were associated with a five-minute increase in surgery time per repetition. Lastly, ROSA®-assisted surgeries following restricted inverse kinematic alignment resulted in an average deviation from mechanical alignment by 1.6° ± 1.9° varus. While we demonstrated accuracy and deviation from the conventional alignment strategy, along with logistic challenges, of the ROSA® Knee System, future studies are needed to investigate its clinical value.

In our retrospective analysis of 71 ROSA® cases, we demonstrated highly accurate planning of resection in three out of four resections. Both accuracy and precision are below previously published levels of 1.0 mm and SDs of 2.2. As compared to literature, the mean difference of the femoral resection was less than 1 mm with a SD < 2mm [4-7,11]. Thereby, it is concluded that ROSA®-assisted TKA is indeed accurate for femoral resection. For the lateral proximal tibia, we observed a discrepancy of 2.5 mm between planning and execution. We believe this discrepancy is mainly because the tibial validation tool sits less stably on the resected proximal tibia when compared to the femoral validation tool. In addition, soft tissue impingement between the validation tool and the resected bone can cause erroneous values. These issues were also communicated to Zimmer Biomet as a potential source of error. Part of the discrepancy between validated values and projected values for both tibia and femur could also be explained by saw deviation on sclerotic bone and by frame jumping, i.e., the error between the transmitter and transducer.

Interpreting results due to possible inappropriate validation might also explain variability in stability assessment. In this study, in which restricted inverse kinematic alignment is aimed for, a medial laxity of up to 2 mm and a lateral laxity of up to 4-5 mm is aimed for. This is checked clinically during surgery, and only when deemed satisfactory, the final components were cemented. However, the clinically assessed gaps can differ from the perioperative gaps as assessed by ROSA®. In our study, this was observed in 50% and 10% medially and laterally, and might also be caused by frame jumping. Additionally, the valgus and varus stress forces given during perioperative measurements may vary, as these forces are imputed by the surgeon and thereby affect the results. Therefore, the robotically measured gaps need to be interpreted with caution. Efforts to reduce variability in applied stress forces and optimize the validation tools might further decrease variability in gap assessment during surgery.

The accuracy of determining implant sizes is high. The observed mismatches mainly highlight the importance of adequate landmark registration. For example, for the femoral component, it is important not to take the reference points on the anterior cortex too far medially or laterally to avoid over- or undersizing of the femoral component. The same holds true for the mapping of the posterior condyles of the femur. Especially in larger femurs, the tool to map the posterior condyles can be too short, resulting in underestimation of posterior condylar offset and translating back into an erroneous smaller femoral size.

A logistical disadvantage of using the ROSA® Knee System is prolonged surgery durations. In line with previous research, ROSA® surgeries took ~20 minutes longer than conventional surgeries, performed by the same surgeons over the same period on comparable patients [14-16]. For surgeries in the proficiency phase, the difference was still ~13 minutes in our study and others [14]. Despite prolonged surgery duration in the ROSA®-assisted TKA, complication rates are similar as compared to conventional TKA.

Our data clearly shows that insufficient resections; hence, requirements for repeating resections are a cause for longer surgery durations. Re-resections during surgery might be due to suboptimal digital planning. Most resections are planned too conservatively, which might result in a tight TKA and subsequently lead to re-resection. Moreover, overly aggressive valgus and varus stress could result in an artificial big gap assessment. The ROSA® Knee System Software will use these results to determine the needed resections, resulting in a too-tight TKA. We nowadays plan resections as if an 11 mm poly would be used. In our experience, this leads to a well-balanced knee with a 10 mm poly in the definitive implant. Obviously, being too conservative at the risk of required repetition is less favorable than too progressive resection, which can be corrected by a larger insert.

Limitations

Several limitations were identified in this study. First, the study is retrospective in nature, and therefore, selection bias is possible. The study is not classically powered, and the sample size is small, but the number of included patients is almost comparable to the prospective study performed by Rossi et al. [4]. Moreover, there were no post-operative full leg weight-bearing radiographs or CT scans to compare the measured alignment with the planned and validated alignment. In addition, patients within the learning curve are included in the study and could interfere negatively with the findings of this study. However, when comparing surgeries during the learning phase to surgeries in the proficiency phase, no differences were found. Lastly, this study lacked a long-term follow-up and clinical relevance; further research is necessary to determine this.

## Conclusions

ROSA®-assisted primary TKA deviated from mechanical alignment into a more kinematic alignment. A high degree of accuracy in resection measurements is seen for femoral components, yet less for tibial components. High accuracy is seen in predicting implant sizes when using ROSA®-assisted primary TKA. Some measures of low accuracy warrant further investigation into the validation tools of the ROSA® Knee System. In further research, the shift to a more functional alignment and the high accuracy in predicting implant sizes and resections need to be linked to functional outcome data to make meaningful interpretations possible on targets to be achieved. As such, we eagerly anticipate the results of our ongoing randomized controlled trial, assessing the clinical value of ROSA®-assisted TKA as compared to conventionally placed TKA.
